# Hybrid Repair Combined with Fresh Arterial Allograft Extra-Anatomical Reconstruction: The Treatment of Infrarenal Abdominal Aneurysm above an Aortobifemoral Bypass Complicated by an Infected Pseudoaneurysm in the Left Groin

**DOI:** 10.1155/2020/8819305

**Published:** 2020-11-07

**Authors:** Robert Novotny, Tomas Marada, Jiri Novotny, Jakub Kristek, Jaroslav Chlupac, Michal Kudla, Kvetoslav Lipar, Jiri Mendl, Jiri Fronek, Libor Janousek

**Affiliations:** ^1^Transplant Surgery Department, Institute for Clinical and Experimental Medicine, Prague, Czech Republic; ^2^Department of Radiology, Institute for Clinical and Experimental Medicine, Prague, Czech Republic; ^3^Department of Anatomy, Second Faculty of Medicine, Charles University in Prague, Prague, Czech Republic

## Abstract

**Introduction:**

A 72-year-old male patient was admitted into our centre with large infected pseudoaneurysm (PSA) in the left groin. The patient underwent a CT angiography (CTA) that confirmed a large partly thrombosed 6.5 × 5.5 cm PSA in the left groin arising from the distal anastomosis of the aortobifemoral bypass (ABF). Furthermore, the CTA revealed 11 cm juxtarenal abdominal aortic aneurysm (JAAA) from which the proximal anastomosis of the ABF was arising.

**Method:**

Aorto-uni-iliac stent graft Cook was placed from the right groin trough native severely stenotic right iliac arteries with proximal landing zone below the renal arteries, excluding the JAAA and the ABF. The distal landing zone was in the common iliac artery maintaining patent right internal iliac artery. Afterwards, a femoro-femoral crossover bypass from right to left was performed using a fresh arterial allograft. Postprocedurally, the hospital stay was uneventful. The left groin PSA cultures came positive for S*taphylococcus epidermidis* and *Corynebacterium tuberculostearicum*, both sensitive to vancomycin and rifampicin.

**Result:**

The patient underwent intravenous ATB treatment with vancomycin for two weeks, followed by four weeks of oral rifampicin. The patient was discharged on the 20^th^ postoperative days.

**Conclusion:**

Hybrid repair combining aortic stent graft and extra-anatomical bypass in the treatment of infected distal parts of an aortofemoral bypass is an acceptable treatment modality.

## 1. Introduction

Prosthetic aortic graft infection (AGI) remains a feared complication by all vascular surgeons. Indicators predicting AGI are difficult to compare. The main reasons are the definition of AGI, case selection, and follow-up. Thus, the reported incidence varies between 0.5% and 6% with mortality ranging from 10% to 43% [1]. AGI had been traditionally treated by an intense intravenous (i.v.) antibiotic treatment, infected graft removal, debridement, and revascularization using extra-anatomic bypass or in situ reconstruction [2]. Early AGI (<3 months after surgery) mainly involves the removal of the entire grafts, as the graft is not fully incorporated. Late AGI presentation varies from a femoral artery pseudoaneurysm (PSA) to an anastomotic disruption potentially complicated by life-threatening bleeding [3]. In the era of advanced endovascular devices, a hybrid approach can be used in patients with late AGI reducing patient's morbidity and mortality [4].

## 2. Case Report

**Figure 1:**
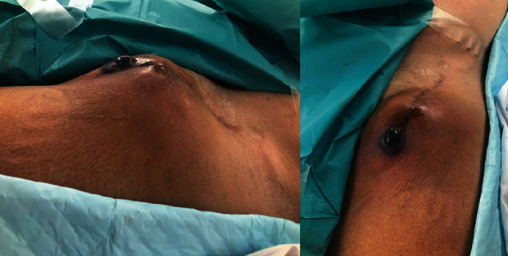
Infected pseudoaneurysm in the left groin.

**Figure 2:**
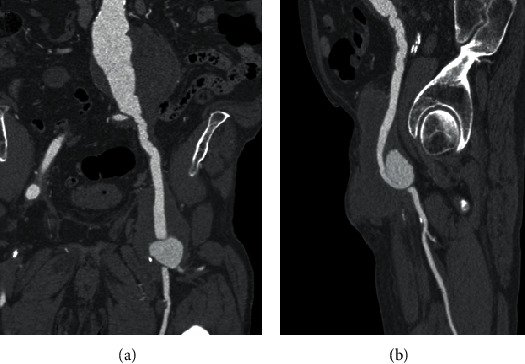
Patient's CT angiography on admission: (a) asymptomatic 11 cm juxtarenal abdominal aortic aneurysm with partially thrombosed 65 × 55 mm pseudoaneurysm in the left groin; (b) partially thrombosed 65 × 55 mm pseudoaneurysm in the left groin.

A 72-year-old male patient was admitted into our centre with large infected pseudoaneurysm (PSA) in the left groin (CRP: 209.4 mg/L; WBC: 7.7 × 10^9^/L) (Figure 1). Patient's medical history revealed the following: aortobifemoral bypass (ABR) 2006, resection of the ABF's left branch for suspected infection at a different centre 2010, and hyperlipoproteinemia. Patient's only comorbidity was compensated arterial hypertension (WHO stage 1). The patient underwent a CT angiography (CTA) that confirmed a large partly thrombosed 6.5 × 5.5 cm PSA in the left groin arising from the ABF distal anastomosis (Figure 2(a)). Furthermore, the CTA revealed 11 cm juxtarenal abdominal aortic aneurysm (JAAA) from which the proximal anastomosis of the ABF was arising (Figure 2(b)). Based on the CTA results, the patient was planned for a hybrid treatment: aorto-uni-iliac stent graft combined with femoro-femoral crossover bypass using an arterial allograft (AA). The patient was immediately started on an empiric i.v. antibiotic therapy (ATB). The patient received a daily dose of *amikacin* 3.6 g*+ampicillin* 3 g*+clindamycin* 1.8 g. The patient was placed on a waiting list for arterial allograft under an “urgent” status. Fresh AA (common and external iliac artery) was available within five days.

**Figure 3:**
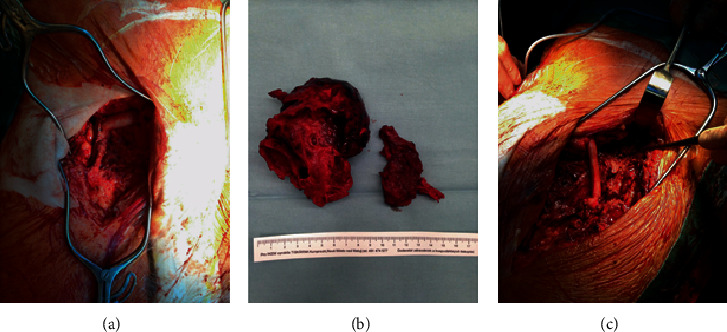
Perioperative finding. (a) Right groin: end to side anastomosis of arterial allograft on the common femoral artery. (b) Resected thrombosed pseudoaneurysm from the left groin. (c) Left groin: end to side anastomosis of arterial allograft on the common femoral artery bifurcation. Ligated common femoral artery below inguinal ligament (arrow).

The procedure was carried out under full anaesthesia. Fist, aorto-uni-iliac stent graft Cook (Cook Ink, Bloomington, USA) was placed from the right groin with proximal landing zone below the renal arteries excluding the JAAA and the ABR. The stent graft was placed trough native severely stenotic right iliac arteries. The distal landing zone was in the common iliac artery above the iliac bifurcation maintaining patent right internal iliac artery. Afterwards, a femoro-femoral crossover bypass from right to left was performed using fresh AA. An anastomosis on the right common femoral artery was performed using polypropylene 6/0 (Figure 3(a)). The AA was subfascialy carried over to the left groin, where massive infected PSA was resected (Figure 3(b)). The proximal part of the common femoral artery was ligated below the inguinal ligament. Femoral bifurcation was dissected, and the AA was anastomosed to the femoral bifurcation using polypropylene 6/0, restoring blood flow to the superficial and deep femoral arteries (Figure 3(c)). Cultures were taken from the left groin PSA and resected left branch of the ABR.

Postprocedurally, the hospital stay was uneventful. The patient was immediately started on *tacrolimus*. The empiric antibiotic treatment was continued until the results of the cultures were available. The ABF left branch cultures came negative. The left groin PSA cultures came positive for S*taphylococcus epidermidis* and *Corynebacterium tuberculostearic*, both sensitive to vancomycin and rifampicin. The patient ATB treatment was changed to i.v. vancomycin for two weeks, followed by four weeks of oral rifampicin treatment.

The patient was discharged on the 20^th^ postoperative days with patent AA bypass, low infection parameters (CRP: 27.2 mg/L; WBC: 7.7 × 10^9^/L), *tacrolimus* daily dose of 9 mg (3.8 *μ*g/L), and 100 mg daily dose of aspirin.

During the first follow-up of six weeks after discharged, the CT showed patent AA bypass and stent graft. Six months after the procedure during the second follow-up, the Doppler ultrasonography showed complete asymptomatic occlusion of AA bypass. The patient was newly diagnosed with prostate cancer. Due to asymptomatic occlusion and new oncologic diagnosis, no further action was taken in order to restore the AA bypass patency. *Tacrolimus* was removed from the patient's medication. Nine months after the procedure, the patient underwent high amputation of left leg complicated by wound dehiscence; non-ST-elevation myocardial infarction was treated conservatively.

## 3. Discussion

The treatment of any vascular graft infection is a complex and challenging process. The treatment of AGI is additionally complicated by the removal of graft from the abdomen and groins in cases of ABR infection. The incidence of ABR infection varies within the reported literature as there is still no solid definition of AGI. The latest study by Bergen et al. reported the 30-day incidence of 1.6% (95% CI 0.4–2.8%), 1-year incidence of 3.6% (95% CI 1.7–5.5%), and 2-year incidence of 4.5% (95% CI 2.4–6.6%) [1]. Variable symptoms of ABR infection based on the time frame from the initial procedure are presented by different clinical manifestations [5]. Currently, there is only one published study by Vogel et al. reporting the AGI incidence corresponding to a time frame. In his study, Vogel et al. reported a 2-year incidence rate of 0.19% (95% CI 0.12–0.26%) on 13,902 patients [6].

Surgical treatment of AGI is a gold standard in younger patients. However, not every patient is physically capable of undergoing such a procedure especially in cases where surgical intervention in the abdomen has to be performed (e.g., suprarenal aortic clamping) [7]. Nonsurgical treatment continues to be the only option for patients with AGI due to their comorbidities. Argyriou et al. showed that conservatively treated AGI has 30-day mortality up to 63% [8]. Lyons et al. showed that AGI caused by infectious agents with low virulence managed purely on antibiotic therapy has a patient's survival over two years [9].

With the advancements in aortic stent grafts and endovascular techniques, hybrid repair in the treatment of an AGI infection is becoming more favourable treatment modality for patients where standard open repair is too risky. The added benefit of hybrid repair is the possibility to address further developing the aortic disease if needed. Hybrid procedures have lower periprocedural mortality and are less surgically invasive when compared to a standard open repair [4, 10].

Vascular reconstruction using antibiotic-impregnated prosthetic grafts is an option in the treatment of AGI only in patients without severe local infection. This applies for patients with suspected ABF infection in the distal anastomosis [2, 11]. If autologous grafts are not available in the treatment of AGI, fresh or cryopreserved arterial allografts are used, as infectious agents can cause a catastrophic graft disruption. Even though arterial allografts maintain reasonable patency, they are compromised by a higher risk of stenosis requiring endovascular interventions [12].

## 4. Conclusion

Hybrid repair combining aortic stent graft and extra-anatomical bypass in the treatment of infected distal parts of an aortofemoral bypass is an acceptable treatment modality for high-risk patients which are unable to undergo a standard open repair where surgical intervention in the abdomen has to be performed.

## Data Availability

Data are available from the corresponding author by request.
